# Primary Immune Thrombocytopenia: Novel Insights into Pathophysiology and Disease Management

**DOI:** 10.3390/jcm10040789

**Published:** 2021-02-16

**Authors:** Anurag Singh, Günalp Uzun, Tamam Bakchoul

**Affiliations:** 1 Institute for Clinical and Experimental Transfusion Medicine (IKET), University Hospital of Tuebingen, 72076 Tuebingen, Germany; anurag.singh@med.uni-tuebingen.de; 2 Centre for Clinical Transfusion Medicine, University Hospital of Tuebingen, 72076 Tuebingen, Germany; guenalp.uzun@med.uni-tuebingen.de

**Keywords:** immune thrombocytopenia, bleeding, platelets, platelet destruction, immune tolerance, megakaryocytes, ITP treatment

## Abstract

Immune thrombocytopenia (ITP) is an autoimmune disorder defined by a significantly reduced number of platelets in blood circulation. Due to low levels of platelets, ITP is associated with frequent bruising and bleeding. Current evidence suggests that low platelet counts in ITP are the result of multiple factors, including impaired thrombopoiesis and variations in immune response leading to platelet destruction during pathological conditions. Patient outcomes as well as clinic presentation of the disease have largely been shown to be case-specific, hinting towards ITP rather being a group of clinical conditions sharing common symptoms. The most frequent characteristics include dysfunction in primary haemostasis and loss of immune tolerance towards platelet as well as megakaryocyte antigens. This heterogeneity in patient population and characteristics make it challenging for the clinicians to choose appropriate therapeutic regimen. Therefore, it is vital to understand the pathomechanisms behind the disease and to consider various factors including patient age, platelet count levels, co-morbidities and patient preferences before initiating therapy. This review summarizes recent developments in the pathophysiology of ITP and provides a comprehensive overview of current therapeutic strategies as well as potential future drugs for the management of ITP.

## 1. Introduction

Primary immune thrombocytopenia (ITP) is a haematological autoimmune disorder characterised by bleeding and a low platelet count of less than 100 × 10^9^/L [1,2,3,4]. There are several factors contributing to the onset of ITP, and the exact mechanisms behind how host immune response turns against own system (autoimmunity) and leads to ITP are still incompletely understood. There is growing evidence suggesting that the main event during ITP is a misbalanced interaction between effectors and regulatory immune cells [5]. This lack of an equitable response leads to a distorted immune tolerance, resulting in increased platelet clearance by immune cells, as well as an impairment in thrombopoiesis. Earlier studies suggested that a low platelet count is largely a consequence of anti-platelet antibodies opsonizing the cells and hence an increased clearance from the circulation [6,7,8]. However, lately, it has been demonstrated by many researchers that cytotoxic T cells also play a vital role in ITP pathomechanism by impairing megakaryopoiesis.

During ITP, it has been observed that although brief, spontaneous remissions can occur frequently in children. On the other hand, adult patients rather display a more chronic form of ITP that correlates with significant clinical presentations including bleeding disorders, haemorrhages in skin or mucous membranes, namely purpura, petechiae and rarely intracranial manifestations of the disease [9,10]. Treatment strategies for ITP are mostly prescribed on the basis of clinical symptoms of the patients with a focus on reducing the risk of severe bleeding, and they do not essentially include the boosting of platelet numbers. As per the guidelines of International Working Group [2,11], patients with acute ITP and without a history suggesting severe bleeding risk are advised to be managed with observation strategy (wait and see). On the other hand, ITP patients require urgent treatment if they are prone to a higher risk of bleeding or carry a severe case of chronic thrombocytopenia.

In this review, we discuss the pathomechanisms that lead to platelet destruction in ITP with a particular focus on recent findings regarding various diversifications during thrombopoiesis. Furthermore, we will provide a broad overview regarding various management strategies of ITP patients. We also outline different treatment options including efficacy and safety of therapeutic medicaments, management of bleeding emergencies as well as a summary of different approved drugs as well as drugs under clinical trials for ITP treatment.

## 2. Pathophysiology of ITP

**Figure 1:**
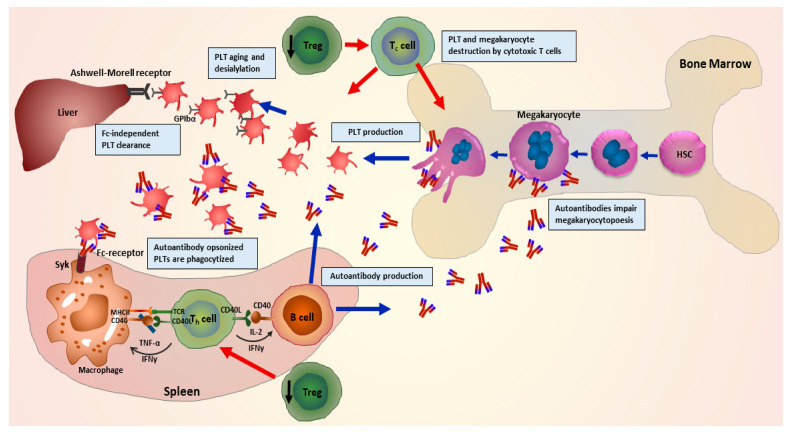
Graphical representation of the pathophysiology of immune thrombocytopenia (ITP) illustrating involvement of multiple immune cells. Impairment of regulatory T cells leads to a disruption in regulation of helper T cell-mediated activation of B cells. B cells in turn produce autoantibodies in abundance leading to opsonisation, phagocytosis and complement activation, desialylation and finally destruction of platelets. Autoantibodies further hinder megakaryocyte maturation (megakaryocytopoiesis), and autoreactive cytotoxic T cells destroy megakaryocytes and platelets. (Adapted from Kashiwagi et al. 2013 [18]).

One of the crucial steps during pathophysiology of ITP is described as the loss of immunological tolerance to autoantigens on patient’s own platelets [12]. Many studies demonstrate that during ITP, a dysregulated T-cell response leads to a distorted balance of helper T cells (Th1/Th2) ratio [13,14], and imbalance further leads to an enhanced number as well as hyperactivity of cytotoxic T cells. Subsequently, this enhanced activity of cytotoxic T cells results in an increase in platelet destruction, combined with improved survival of B cells. An enhanced survival rate of B cells hence facilitates a larger production of autoantibodies, leading to an accelerated rate of platelet clearance. Autoantibodies opsonize platelets leading to enhanced phagocytosis, apoptosis, complement activation and impaired thrombopoiesis [15,16,17] (Figure 1).

Although platelet destruction in the spleen primarily involves constant fragment (Fc)-dependent mechanisms, various researchers have also described novel mechanisms independent of Fc-mediation [19,20,21]. In a study, it was shown that ITP-autoantibodies can induce glycan modifications on platelet surface glycoproteins (GPs). Upon further recognition by Ashwell–Morell receptors which are expressed on hepatocytes, this GPs modification leads to accelerated platelet clearance in the liver [22]. CD8+ T cells from ITP patients also induce platelet desialylation and platelet phagocytosis by hepatocytes [23]. This might explain a potential mechanism how splenectomy remains ineffective in some ITP patients. In an intriguing retrospective study with a cohort of 61 ITP patients, it was shown that platelet desialylation and subsequent reduction in response to first line of treatments was independent of any Fc-mediated mechanism [24].

A recent study by Quach and colleagues demonstrated that ITP patients who did not respond to therapy were more likely to produce autoantibodies against the ligand binding domain (LBD) of GPlb/lX [25]. This specific binding leads to activation of GPIb/IX via crosslinking of platelet receptors and unfolding of a mechanosensory domain and platelet destruction, providing further a pivotal evidence of Fc-independent mechanism [25]. Recently, we demonstrated that novel effector functions of autoantibodies in ITP modulate the disease and might interfere with the clinical outcome for patients. We showed that a subgroup of autoantibodies induces cleavage of sialic acid residues from the surface of human platelets and megakaryocytes during ITP. Furthermore, autoantibody-mediated desialylation was found to interfere with the cell–extracellular matrix protein interaction and hence leading to impaired platelet adhesion and megakaryocyte differentiation [26]. This hints towards a potential use of sialidase inhibitors as a treatment approach in combination with other therapies to boost platelet numbers in some patients who have failed to respond to previous therapies.

It is well established that intrinsic apoptotic pathway plays a significant role in platelet life cycle. Many research groups have demonstrated the role of ITP-autoantibodies in regulating platelet apoptosis and pathways involved. There is ample evidence showing that various apoptosis markers including phosphatidylserine (PS) exposure, depolarisation of the mitochondrial transmembrane potential, Bcl-2 family protein expression, activation of caspase-3 as well as of caspase-9 are significantly involved in platelet apoptosis in ITP [27,28]. Immunoglobin infusion was shown to successfully mitigate platelet apoptosis in adult as well as paediatric patients [29,30]. Interestingly, it was shown that apoptotic platelets were not found in ITP patients harbouring anti-GPIa/IIa autoantibodies but only in those who carried anti-GPIIb/IIIa and anti-GPIb autoantibodies [31], indicating a potential role of autoantibody specificity.

Autoantibodies produced during ITP not only affect platelet survival but also platelet formation by megakaryocytes [32]. It has been shown that autoantibodies bind and hinder the megakaryocyte maturation, resulting in reduced platelet formation [33,34]. It was demonstrated in vitro, that autoantibodies inhibit platelet production by impairing megakaryopoiesis and maturation [35,36,37]. However, the role of megakaryocyte apoptosis still needs to be investigated in terms of involvement in the pathophysiology of ITP. There have been some hints and contradicting claims through results generated via earlier and recent investigations. A study in fact demonstrated that treatment with ITP plasma rather leads to a reduced apoptosis of megakaryocytes [38]. Haematopoietic stem cells (HSCs) isolated from healthy umbilical cord blood were co-cultured with plasma of ITP patients, resulting into a decrease in apoptosis, reduced expression of tumour necrosis factor-related apoptosis inducing ligand (TRAIL) and increased expression of the anti-apoptotic protein Bcl-xL in differentiated megakaryocytes [39]. On the other hand, in contrast to these findings, an earlier in vivo study suggested that megakaryocytes in fact undergo enhanced apoptosis in the presence of autoantibodies [40]. It was observed in biopsies of ITP patients that increased apoptosis involves nuclear fragmentation, chromatin condensation and activation of caspase 3. This further leads to phagocytosis of the polyploid cells by resident macrophages in the bone marrow [40]. Another recent study showed that an increased megakaryocyte apoptosis occurs in the bone marrow samples obtained from ITP patients [41].

## 3. Clinical Manifestations

The overall annual incidence rate of ITP is 1.6–5.3 per 100,000 persons, and it is more frequent in women than men [42,43,44]. ITP can be classified according to disease duration as acute (<3 months), persistent (3 to 12 months) or chronic (>12 months). Compared to children, adults are more likely to develop chronic ITP disease. While up to 60% of adults develop chronic diseases [45,46], only 20–30% of children have persistent thrombocytopenia at 12 months [47,48].

Most patients are presented with bleeding symptoms such as petechiae, purpura, haemorrhages of the mucous membranes of the mouth and nose, urogenital bleeding or increased menstrual bleeding [49]. Some patients can be asymptomatic at presentation and 30–40% of patients with chronic ITP do not have any bleeding symptom [50]. Bleeding risk is calculated as 8% per year in ITP patients [51].

Major bleedings are associated with a high rate of mortality [52,53]. Reported rates of severe bleeding vary depending on the population studied. In a recent literature review including 108 studies reporting on 10,908 patients, the weighted proportion for intracerebral haemorrhage (ICH) was 1.0% (95% CI, 0.7–1.3) and for non-ICH severe bleeding was 15.0% (95% CI, 9.3–21.8) [54]. Forsthye and colleagues reported a severe bleeding episode that required rescue medication (intravenous immunoglobulin, corticosteroid injections or platelet transfusions) in 10,2% of adult ITP patients within 6 months after starting therapy with thrombopoietin receptor agonists (TRO-RA) [55]. In a retrospective evaluation of the McMaster ITP registry, Arnold et al. found that 56% of ITP patients experience clinically significant bleeding at some point during their disease course and 2.2% had ICH [56].

Compared to ITP patients with normal platelet counts, those with a platelet count between 25 to 49 × 10^9^ /L and <25 × 10^9^ /L had 2.4 fold and 4.5 fold increased bleeding rates, respectively [51]. Furthermore, bleeding requiring a hospital contact within 1 year prior to ITP diagnosis was associated with a 3-fold increased rate of subsequent bleeding [51]. The use of non-steroidal anti-inflammatory drugs (NSAIDs) was found to be associated with any bleeding (OR 4.8, 95% CI 1.1–20.7) and anticoagulant drugs were associated with severe bleeding (OR 4.3, 95% CI 1.3–14.1) [57]. In a large patient cohort, Hato et. al. found that age (>60 years), platelet count (<10 × 10^9^ /L), and the presence of haematuria are associated with increased risk for ICH [58].

Fatigue is common in patients with ITP, and its impact on health-related quality of life in ITP patients has been until recently underappreciated [59]. Treatments that increase platelet count also reduce fatigue [60,61]. However, it is also recommended to use treatment strategies that directly target fatigue to improve the health-related quality of life in ITP patients [62].

Paradoxically, an increased frequency of thromboembolic events has been reported in ITP patients [63,64]. Therefore, it is crucial that ITP patients should be aware of the risk of thromboembolic events. Patients should be educated that ITP can increase not only the risk of bleeding but also the risk of venous and arterial thromboembolism [50]. Furthermore, patients at risk of embolic events should be followed more closely. Presence of lupus anticoagulants is related to thrombotic events [65]. The increased levels of prothrombotic, platelet-derived microparticles and complement activation on antibody-coated platelets also contribute to the development of thrombosis in ITP [66]. In addition to disease and patient related factors, ITP treatments such as TPO-RA and splenectomy could also increase the individual risk of thromboembolic events [64,67,68]. Clinical management of thrombocytopenic patients who require anticoagulant or antiplatelet therapy due to cardiovascular comorbidities is a serious challenge. An aggressive treatment of ITP may be required in these patients to achieve a safe platelet count over 50 × 10^9^L [50].

The overall mortality rate is slightly higher than general population in ITP patients, predominantly due to increased cardiovascular disease, infection, bleeding and haematological cancer related mortalities [69].

## 4. Diagnosis

ITP is usually diagnosed after precluding other potential causes of thrombocytopenia. A diagnosis is performed in patients with a low platelet count (<100 10^9^/L) with no evidence or history of an underlying condition, which can lead to thrombocytopenia, including a physical examination, evaluation of blood counts and visual examination of blood smears. However, since thrombocytopenia may be a multifactorial condition, it is indeed complicated to identity substitute causes, and examining physician needs to have a broad knowledge in platelet disorders. A confirmation of ITP is achieved via detection of characteristic platelet-specific autoantibodies, free in patient serum or bound to own platelets [70]. As per recommendations of various regulatory guidelines, GP-specific assays, for example direct monoclonal antibody immobilisation of platelet antigens (MAIPA test) or direct immunobead assays prove the diagnosis of ITP, and further laboratory tests are deemed unnecessary [71]. However, current ASH-guidelines of 2019 do not give any clear recommendations for antibody evaluation in ITP patients, as there is still lack of strong evidence supporting clinical advantage of the assays [4]. We strongly recommended that as a part of initial assessment, presence of platelet autoantibodies should be evaluated. A positive test result at this stage establishes a sound basis for further diagnostic procedures and paves ways for initiating the treatment. It is notable to mention that although GP-specific tests have shown an excellent specificity, the lack of sensitivity is an important issue to consider. The low sensitivity of the test can often produce negative results, and care needs to be taken while interpretation and subsequent recommendation. Other potential hurdles in implementing antibody testing as a part of mandatory diagnostic regime for ITP also include unavailability of experienced staff, equipment and set up, as well as cost effectiveness.

Therefore, it is recommended to establish an appropriate diagnostic set up to analyse ITP during early phase of patient examination.

## 5. Treatment of ITP

**Figure 2:**
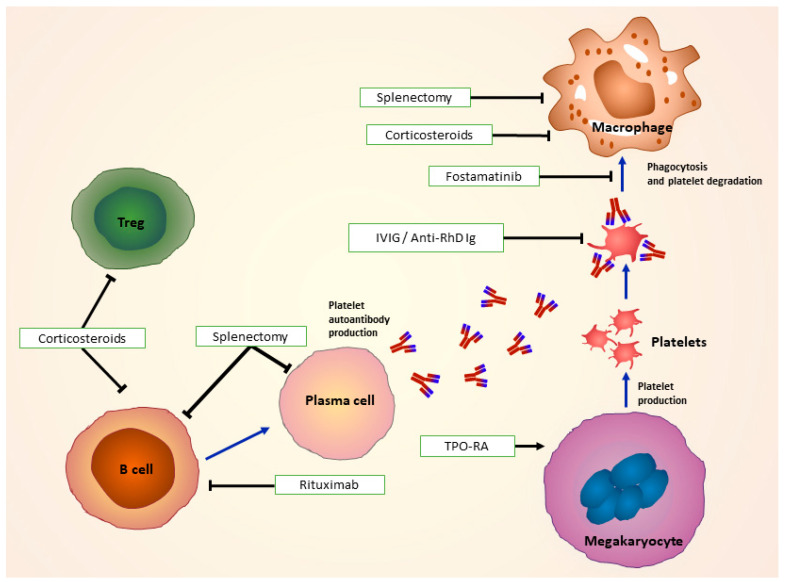
Treatments for immune thrombocytopenia: Corticosteroids used as first line of treatment modulate Treg, B-cell and FcR function. In combination with or without IVIg and anti-D, they impair antigen presentation and recognition of autoantibody-coated platelets by macrophages. Second-line treatments such as surgical splenectomy remove spleen as a site of platelet destruction, and drugs as rituximab target antibody-producing B cells. TPO-RAs, such as romiplostim and eltrombopag work by stimulating platelet production by megakaryocytes. Fostamatinib impairs Syk-mediated phagocytosis of platelets. FcR: Fc receptors; IVIg: Intravenous immunoglobulin; TPO-RA: Thrombopoietin receptor agonist; Syk: spleen tyrosine kinase. (Adapted from Newland et al. 2018 [72]).

The main goals of ITP treatment are to intervene in the case of an acute severe bleeding and to prevent future bleeding events (Figure 2).

### 5.1. First-Line Treatments and Treatment of Bleeding Emergencies

The decision to start a treatment in newly diagnosed ITP depends on several factors. Current guidelines recommend a platelet count of 20 to 30 × 10^3^/µL as a cut-off value to start intervention in adult ITP patients [4]. Other than thrombocyte count, patient related factors can help to determine the risk of bleeding such as age (e.g., >65 years), previous bleeding events, comorbidities associated with high bleeding risk (i.e., hypertension, cerebrovascular disease), renal or hepatic impairment, medication with anticoagulants and platelet inhibitors, surgical interventions and risky life style (i.e., combat sports) [51,57,73]. A higher platelet count (>50,000 µL) should be considered for these patient populations.

As emphasized in the current guidelines, the decision regarding ITP treatment should be made in agreement between physician and patient. The patient should be informed about the benefits and possible side effects of treatment options. It should be considered that some side effects of treatments might pose a greater risk for the patient than ITP itself [74]. Advantages and disadvantages of ITP treatments are summarized in Table 1.

#### 5.1.1. Glucocorticoids

Glucocorticoid treatment is the most-commonly used first-line therapy in patients with ITP [43,75]. The beneficial effects of glucocorticoids include reduction of platelet clearance by reticuloendothelial system [76,77]. Platelet count usually increases within a couple of days after therapy initiation [49]. Two most-commonly used glucocorticoids are prednisone (1 mg/kg orally per day for 2–3 weeks, with a gradual withdraw and discontinuation by 6 to 8 weeks) and high-dose dexamethasone (one or more cycles of 40 mg orally, once daily for 4 days, usually 4 weeks apart) [78]. Current ASH guideline recommends against the use of glucocorticoids longer than 6 weeks [4]. On the other hand, some others suggested that a longer low-dose steroid therapy could be considered to keep the platelet counts over 30 × 10^3^/ml if a response with initial steroid therapy has been achieved [11]. Several studies demonstrated more rapid response with dexamethasone as compared to prednisone, but overall response rates are not significantly different in the long term after 6 and 12 months [79]. Similarly, Wang et al. reported a rapid response with high-dose dexamethasone compared to prednisolone, but sustained response rates were similar at 12 months and later [78]. Of note, dexamethasone seems to have a better safety profile (fewer Cushing’s disease, weight gain and infection rates) in comparison to prednisolone [80].

Despite the high early-response rate, most of the patients do not have a sustained response after the cessation of glucocorticoids. In fact, approximately 80% of patients respond initially to corticosteroids, but only 20 to 40% of these patients achieve sustained response when steroids are discontinued [81,82]. As a predictive factor, Wang et al. measured anti-platelet antibody levels in ITP patients under glucocorticoid treatment [78]. They found that presence of anti-GPIb-IX antibodies predicts a poor initial response to corticosteroids [78]. Further studies are needed to determine the role of antiplatelet antibodies in predicting the corticosteroid response.

It is crucial to closely monitor the patients for possible side-effects of glucocorticoids such as hypertension, hyperglycaemia, sleep and mood disturbances, gastric ulceration, myopathy, glaucoma and osteoporosis [4]. To prevent severe toxicities, corticosteroids should be tapered appropriately and discontinued in non-responding patients. Non-responders and patients with contraindication to steroid therapy such as (pregnancy, diabetes mellitus, active infection and psychiatric disorders) can be treated with other first-line treatments-IVIG and IV anti-D [4].

To increase the rate of sustained response, combination of dexamethasone with second line treatments such as rituximab have been investigated. A recent meta-analysis compared the effectiveness of the combination of high-dose dexamethasone and rituximab with dexamethasone alone in ITP [83]. Overall response rate at month 3 (RR = 5.07, 95% CI: 2.91–8.86, and *p* < 0.00001) and sustained response rate at 12 months (RR = 1.73, 95% CI: 1.36–2.91, and *p* < 0.00001) was significantly higher in combination arm than that in monotherapy. Furthermore, the rate of adverse events has not significantly increased with combination therapy [83].

#### 5.1.2. Intravenous Immunoglobulin (IVIG)

IVIG has been introduced into the treatment of ITP in 1980s [84]. IVIG is prepared by purification from the pooled plasma of healthy donors [84]. It contains polyvalent IgG (80 to >95%) and irrelevant amount of IgA and IgM. IVIG is thought to inhibit Fc-mediated phagocytosis of antibody coated platelets by reticuloendothelial system [85]. Platelet count usually increases within 48 hours after IVIG application [86]. The preferred treatment regime is 1 g/kg per day, which should be repeated for two consecutive days [2]. A lower dose of 0.2–0.4 g/kg/day can also be used for 4–5 days [87]. In a meta-analysis of 13 randomized studies, low dose IVIG regimes were found to be as effective as high dose IVIG, and low-dose-IVIG was associated with a significantly reduced risk of side-effects (OR = 0.39 (95% CI = 0.18–0.83) [88].

Limited number of randomized controlled studies compared the effectiveness of IVIG and corticosteroids as a first line therapy in ITP in adults [89,90]. Godeau et al. demonstrated that IVIG increases platelet count more effectively than high-dose methylprednisolone in adults with newly diagnosed ITP (79% vs. 60% response rate) [90]. In a smaller study, adult ITP patients were treated with oral prednisone (1 mg/kg/day; *n* = 17), high-dose IVIG (400 mg/kg on days 1 through 5; *n* = 13) or a combination of both agents (*n* = 13). A platelet response (>50 × 10^9^/L) was achieved in 82%, 54% and 92% of patients, respectively [89].

There may be a relationship between the presence of anti-platelet antibody and the response to IVIG. Peng et al. found that the response rate was significantly higher in patients without anti-GPIb-IX autoantibodies compared to those with anti-GPIb-IX autoantibodies (80.0% vs. 36.4%), while the presence of the anti-GPIIb/IIIa autoantibodies had no effect on response to treatment [91]. However, others failed to show a significant relationship between an autoantibody and nonresponse to IVIG [92].

Most frequent adverse effects of IVIG include headache, pyrexia and vomiting [93]. Severe side effects such as acute kidney injury, aseptic meningitis and thrombotic events are rare [94].

#### 5.1.3. Anti-RhD Immunoglobulin (Ig)

Anti-RhD consists of IgG selectively taken from the plasma of donors immunized to the Rhesus D antigen [85]. Anti-RhD Ig binds to Rh-positive erythrocytes and these antibody-coated erythrocytes competitively inhibit the destruction of antibody-coated platelets by binding and occupying Fc receptors on phagocytes in the spleen [95]. Anti-RhD is therefore only effective in Rh-positive patients with an intact spleen. A single intravenous dose of 50 to 75 µg/kg is recommended [96]. A safe subcutaneous administration in small children or patients is also described in the literature [97]. Side effects include mild infusion reactions such as headache, nausea, chills, fever and mild to moderate haemolysis [98]. However, life-threatening episodes of severe intravascular haemolysis and disseminated intravascular coagulation after Anti-RhD Ig administration have also been reported [99,100]. These reports led to the withdrawal of an Anti-RhD product (WinRho^®^ SDF, Cangene Europe Ltd, London, UK) from European markets in 2009 [50].

### 5.2. Treatment of Active Bleeding

In case of clinically relevant bleeding, glucocorticoids, IVIG and platelet transfusion are used alone or in combination to increase the platelet count rapidly [11]. Besides, other interventions such as endoscopy or surgery may be necessary depending on the severity and the site of the bleeding [52]. Furthermore, anticoagulant and antiplatelet medications should be ceased immediately, if possible. Since the effect of platelet transfusion is limited due to rapid clearance of platelets by the circulating autoantibodies, combining platelet transfusion with IVIG or corticosteroids might be useful [11]. Although IVIG increases platelet count in most of the cases within 48 hours, its effect is temporary, and platelet count decreases after 1 to 2 weeks. Therefore, concomitant use of glucocorticoids with IVIG can be considered to achieve a more sustained response than that with IVIG alone [90]. Of note, the recommendations for the treatment of active bleeding in ITP are based on small observational studies, and randomized controlled studies are urgently needed.

The Updated International Consensus Report recommends the use of TPO-RA in the case of a life-threatening bleeding if initial treatments with corticosteroids, IVIG and thrombocyte transfusion fails to increase the platelet count [11]. Roumier et al. used high dose romiplostim (10 μg/kg body weight) together with vinca alkaloids in 30 patients with severe bleeding and compared the results with a historical patient group treated with vinca alkaloids only [101]. Both groups constituted of patients who failed to achieve a response after initial therapy with IVIG, corticosteroids and/or platelet transfusion [101]. At day 7, complete response (60% vs. 29%, *p* < 0.05), and at day 14, both partial response (80% vs. 43%, *p* < 0.05) and complete response (70 vs. 17%, *p* < 0.0001) were significantly higher in the romiplostim plus vinca alkaloid group compared to the vinca alkaloid group alone [101]. Although this study shows the effective use of high dose romiplostim in life-threatening bleeding in ITP patients, two patients (6.6%) treated with high dose romiplostim developed major thromboembolic events. Therefore, the risk over benefit ratio should be carefully assessed for each patient.

Antifibrinolytics (tranexamic acid and aminocaproic acid) are successfully used to control significant bleeding in patients with ITP [102,103,104]. Oral contraceptives can be used in female patients with menorrhagia. In life threatening bleeding emergencies, recombinant activated factor VII may be a useful supportive treatment [105,106,107].

### 5.3. Second-line treatments

#### 5.3.1. Thrombopoietin-Receptor Agonists (TPO-RA)

Romiplostim is an Fc-peptide fusion protein and administered as a once-weekly subcutaneous injection. The recommended initial dose is 1 µg/kg per week, which can be adjusted by weekly increments of 1 µg/kg according to platelet response to achieve a platelet count of >50 × 10^9^ platelets/L. The maximum dose is 10 µg/kg/week. Romiplostim is indicated in adult ITP patients who have had an insufficient response to corticosteroids, immunoglobulins or splenectomy. Self-administration of romiplostim by patients can help in reducing healthcare costs and increase patient comfort by eliminating the need to visit the hospital every week for applications [108].

Owing to the effectiveness and safety-profile of TPO-RAs recent studies explored the use of these drugs in other patient groups also. Kuter et al. investigated the effectiveness of romiplostim in patients with ITP for less than 12 months by analysing the data from 9 studies [109]. They found that the number of patients with a platelet response at ≥75% of measurements were higher for romiplostim (74% (204/277)) than for placebo/standard of care (18% (6/34)) in patients with ITP ≤1 year. More importantly the rate of treatment free remission (platelet counts ≥50 × 10^9^ /l for ≥6 months) was higher in patients with ITP ≤ 1 year compared to those with ITP >1 year [109]. Clinically relevant bleeding-related episodes are significantly lower in patients on romiplostim therapy [110,111]. Kuter et al. followed 292 adult ITP patients receiving romiplostim as weekly treatment and observed that the platelet response is maintained with stable dosing for up to 5 years of continuous treatment [67].

Most frequently observed side effects are headache, arthralgia, myalgia, dizziness and insomnia [112]. Thromboembolism and bone marrow fibrosis are the most feared complications of TPO-RA in ITP patients. Gernsheimer reported that romiplostim does not present an increased risk of thromboembolic events compared to placebo [111]. However, close monitoring of patients for thromboembolic events is recommended. Bone marrow changes were observed in a small proportion of patients receiving romiplostim [113]. But the bone marrow fibrosis is reversed after the end of treatment [114,115].

Eltrombopag, which is a synthetic non-peptide molecule, binds selectively with thrombopoietin receptors on megakaryocytes and induces thrombopoiesis [116]. Eltrombopag is recommended for adult ITP patients who have had an insufficient response to corticosteroids, immunoglobulins or splenectomy. Eltrombopag is administered orally as a daily tablet. Daily dose is 25–75 mg according to the age and hepatic function status of the patient. To ensure an adequate absorption of eltrombopag, it should be taken at least 2 hours before or 4 hours after any medications or products containing polyvalent cations (such as antacids, calcium-rich foods and mineral supplements). Many patients have difficulty meeting these dietary requirements and an alternative intermittent dosing 1–5 times weekly have been recommended [117]. Due to the risk of hepatotoxicity, a dose reduction is necessary in patients with hepatic impairment and a close monitoring of liver enzymes and bilirubin every two weeks throughout the treatment is indicated [118].

Randomized controlled studies showed that eltrombopag achieved early platelet response in 70–80% of the patients and a remission rate of 20–30% [119,120,121,122]. In an open-label extension study, 85% of the patients achieved a platelet response, and 52% of them had a continuous response of 25 weeks or longer [123]. Furthermore, the incidence of bleeding episodes in patients receiving eltrombopag decreased from 57% to 16% at 1 year [123]. Although some patients seem to have a prolonged/complete remission after pausing TPO-RA, no prognostic marker is currently available to identify such patients [124]. However, recently, an inverse relation between TPO level and response to eltrombopag or romiplostim has been shown [125]. Patients with a normal baseline TPO level are more likely to benefit from a therapy with these drugs [125].

Forsthye et. al. compared the bleeding related adverse events in patients receiving romiplostim or eltrombopag in a retrospective cross-sectional study. Patients on eltrombopag (*n* = 1617) had significantly fewer bleeding episodes compared to those on romiplostim (*n* = 1140) (7% vs. 14%) [55].

In terms of adverse effects, liver functions, thromboembolism and bone marrow fibrosis have been the areas of concern in the long-term use of eltrombopag [126]. Gastrointestinal symptoms (nausea, vomiting and diarrhoea), mild transaminase elevations and headache are the most commonly observed adverse events in clinical studies [122]. In a prospective safety and efficacy study, thromboembolic events were observed in 6% of patients and hepatobiliary side effects in 15% of patients with a median eltrombopag treatment duration of >2 years [123]. Regular follow-up of patients for these side effects is justified.

Avatrombopag is an orally administered TRO-RA and recently received FDA approval for treatment of resistant ITP in adults. Unlike eltrombopag, avatrombopag can be administered without dietary restrictions. Furthermore, avatrombopag does not require monitoring of liver functions [127]. The phase 3 clinical trial showed a longer median number of weeks with platelet count of 50 × 10^9^/L or higher during the first 26 weeks in patients who received avatrombopag than in those who received placebo [128]. A platelet response (a platelet count ≥30 × 10^9^/L, with at least a two-fold increase in platelet count from baseline and an absence of bleeding) has been observed in 56.3% of the avatrombopag treated patients [128]. The recommended initial dose is 20 mg/day. The doses or dosing frequency should be adjusted individually to maintain platelet count greater than 50 × 10^9^/L. The maximum daily dose is 40 mg [127]. The treatment should be discontinued if a platelet response is not achieved in 4 weeks of avatrombopag therapy at a dose of 40 mg/day. Most common side effects are headache, arthralgia, fatigue and diarrhoea. Further studies are needed to ensure the long-term safety of avatrombopag.

#### 5.3.2. Immunomodulators

Rituximab is an anti-CD20 monoclonal antibody that depletes CD20+ B cells and reduces antiplatelet antibody production directly [129]. Rituximab achieves a significantly higher incidence of complete response at 6 months compared to glucocorticoids or placebo in non-splenectomised ITP patients (46.8% vs. 32.5%) [130]. More than one-half of the responders had their response last for at least 1 year, resulting in a 1-year response rate of 38%. Patel et al. reported a 2-year response rate of 31% and a 5-year response rate of 21% in adults treated with rituximab [131]. Sustained platelet response lasts more than 2 years in 50% of patients who have an initial response to rituximab [131,132]. Low dose rituximab therapy has been recommended to avoid treatment related adverse events. A recent systematic review found an overall response rate of 63% and complete response rate of 44% in ITP patients treated with low-dose (100 mg or 100 mg/m^2^ per week for 4 weeks) rituximab instead of the standard dose of 375 mg/m^2^ per week for 4 weeks [133]. Low dose rituximab has a satisfactory efficacy and safety profile [133]. In a long-term follow-up study (median follow-up of 6 years), median duration of response was longer (17 months vs. 11 months), and splenectomy rate was lower (17.2% vs. 26.4) in rituximab-treated patients. However, 70% of the rituximab-treated patients relapsed within two years after response [134]. Hammond et al. showed that response rate at 2 years was 70% in ITP patients treated with rituximab after unsuccessful splenectomy [135]. Wang et al. have recently demonstrated that a positive ANA test is associated with a better initial response but with an unfavourable long-term outcome in ITP patients treated with rituximab [136].

Rituximab should not be prescribed to patients with evidence of an active or previous HBV infection due to the risk of fulminant hepatitis, and other treatment options should be considered [129]. An increased tendency to minor infections after rituximab therapy has been reported. On the other hand, progressive multifocal leukoencephalopathy seem to be rare [137]. Taken together, due to the lower efficacy and higher complications compared with TPO-RAs [138], rituximab should be avoided as first line therapy and used only if there is high evidence for remission [4].

Fostamatinib is an orally available spleen tyrosine kinase (Syk) inhibitor. Syk-dependent phagocytosis of FcγR-bound platelets plays a role in the pathophysiology of ITP, and fostamatinib inhibits antibody-mediated destruction of platelets [139]. Pooled analyses of two randomized controlled trials demonstrated a response within 12 weeks in 43% of the patients compared to 14% of those receiving placebo [140]. In addition, a sustained platelet count ≥50 × 10^9^/L for up to 24 weeks was observed in 18% of refractory ITP patients compared to 2% of those receiving placebo [140]. In the open label extension study with the patients who had a stable response, 21 (78%) patients had maintained the response for 1 year and 15 (56%) for 2 years [141]. In a post-hoc analysis of the phase 3 and open-label extension study, Boccia et al. observed a higher platelet response rate (≥50 × 10^9^/L) (78% vs. 48%) and lower bleeding events (28% vs. 45%) when fostamatinib was used as a second line therapy as compared to its use as a third-or-later-line of therapy [142]. The recommended initial dose is 100 mg twice daily, and the dose can be increased to 150 mg twice daily, if platelet count has not increased to at least 50 × 10^9^ /L after 4 weeks of therapy. Most common adverse reactions are diarrhoea, hypertension and nausea. A monthly monitoring for hepatotoxicity and neutropenia is recommended [143]. Long-term studies are needed to better understand the efficacy and safety profile of fostamatinib in patients with chronic ITP.

#### 5.3.3. Splenectomy

Spleen is the main site of the autoantibody production and platelet destruction. Splenectomy is long regarded as the gold standard therapy for ITP patients who are unresponsive to corticosteroids [144]. Compared to other treatment options, splenectomy has a higher sustainable response rate [4]. However, with the introduction of new medicaments, splenectomy has lost its place in the treatment of ITP [75,145].

Splenectomy achieves a high rate of durable remissions in 60 to 70% of the patients [146]. The need for the third-line treatment is significantly lower in patients who have undergone splenectomy (20%) compared to patients treated with second-line therapy (39–44%) [147]. Vianelli et al. reported a relapse free survival in 67% of the patients for up to 20 years after splenectomy [148]. However, due to the surgical risks and potential long-term complications, splenectomy is usually reserved to chronic ITP patients who failed to respond to standard medical therapies or when therapies are contraindicated [50,144].

Furthermore, the lack of reliable predictors of response to splenectomy hinders the selection of the patients who will benefit from splenectomy [146]. Revealing the main site of platelet sequestration can help to predict the success of splenectomy. Autologous platelet scanning can be used to detect the site of platelet sequestration, but it is technically challenging and not widely available [149]. Knowledge of desialylation capacity of the anti-platelet autoantibodies might also be helpful to detect Fc-independent clearance of platelets in the liver [22].

Complications associated with splenectomy are post-operative bleeding, infection with encapsulated bacteria, sepsis as well as thromboembolic events in venous and arterial circulation (i.e., coronary artery disease, stroke and chronic thromboembolic pulmonary hypertension) [144]. In a retrospective analysis of medical records, among second line treatments, splenectomy had the highest frequency of deep vein thrombosis and pulmonary embolism [147]. Compared to open surgery, laparoscopic splenectomy has a lower rate of postoperative mortality and morbidity and a shorter hospitalization [146,150]. Moreover, the immediate as well as the persistent risks of venous thromboembolism have been shown to be higher among patients with ITP who have undergone splenectomy as compared those who have not [151,152].

Patient’s age must also be taken into consideration during the selection process for splenectomy. Maria et al. showed that patients age at the time of the surgery predicted the response in children [153]. Older children show a better outcome after splenectomy. Recently, Kwiatkowska et al. showed that age (<41 years) together with (BMI < 24.3 kg/m^2^) and preoperative platelet count (≥97 × 103 mm^3^) are independent prognostic factors for ITP remission after splenectomy [154]. Geriatric patients are prone to surgical complications and an increased relapse has been reported in ITP patients over 60 years [155,156]. Therefore, splenectomy should be implemented as a last resort in elderly patients. Last but not the least; splenectomy should not be performed in the first 12 to 24 months after ITP diagnosis because of the chances of spontaneous remission or disease stabilization [11].

### 5.4. New Drugs under Investigation

Rozanolixizumab is anti-neonatal Fc receptor (FcRn) antibody that reduces plasma IgG levels. In a recent phase 2 study, >50% patients with persistent/chronic primary ITP achieved clinically relevant platelet responses (≥50 × 10^9^/L) by day 8 after a single injection of rozanolixizumab at a dose of 15 and 20 mg/kg [157]. Treatment related mild-to-moderate adverse events have been seen in 15 of 66 (21%) patients, and no serious infections have been reported. A phase 3 study is currently recruiting participants (NCT04224688).

Bortezomib, a proteosom inhibitor, induces apoptosis of long-lived autoreactive plasmocytes and reduces secretion of anti-platelet antibodies. In murine models of ITP, bortezomib eliminated long-lived plasmocytes and alleviated thrombocytopenia [158]. Beckman et al. used bortezomib to treat a 63-year-old female patient who had severe thrombocytopenia and bleeding episodes despite the utilization of several treatments including splenectomy [159]. The patient received bortezomib injections in addition to other treatments, and platelet count increased rapidly after the initiation of bortezomib. The results of the ongoing clinical trials (NCT03443570, NCT04083014) will help us to better define, if any, the role of bortezomib in ITP.

Efgartigimod is an Fc fragment that blocks FcRn. In a recent study, patients with a platelet count <30 × 10^9^/L despite treatment received four weekly intravenous injections of either placebo or efgartigimod, at a dose of 5 mg/kg or 10 mg/kg [160]. Antiplatelet antibody levels reduced 40% or more in 8/12 (66.7%) patients treated with efgartigimod at 5 mg/kg and in 7/10 (70.0%) patients treated with efgartigimod at 10 mg/kg. A platelet response >50 × 10^9^/L on 2 occasions has been achieved in 46.2% of the patients on efgartigimod as compared to 25% on placebo [160]. A Phase 3 Study investigating the safety and efficacy of efgartigimod at a dose of 10 mg/kg is ongoing (NCT04225156).

Decitabine is an inhibitor of DNA methylation and used in the treatment of myelodysplastic syndrome. Considering the possible role of DNA-methylation in the aetiology of ITP [161], decitabine seems to be a potential treatment option. Low dose decitabine promotes megakaryocyte maturation and platelet production in patients with myelodysplastic syndrome and ITP [162,163]. In a prospective open label study, Zhou et. al. showed that an overall response rate of 51% with a median initial response time of 28 days in ITP patients [164]. The sustained response rates at 6, 12 and 18 months were 44.44% (20/45), 31.11% (14/45) and 20.0% (9/45), respectively [164].

## 6. Conclusions

In recent years, ITP guidelines have been updated in the context of improved understanding of the pathophysiology of ITP and evidence supporting newly introduced treatments. Despite recent developments, the expected increase in the success rate of treatments has not been achieved yet. A substantial number of patients either do not respond at all or respond only transiently to many treatment interventions. The use of different treatment regimens targeting different key points in the pathophysiology of the disease may increase the success rate. In addition, the development of patient-specific testing methods, which can predict treatment success, may assist in avoiding complications, wasted time and associated costs from unnecessary treatments.

The management of ITP during ongoing 2019 coronavirus disease (COVID-19) pandemic has emerged as an additional challenge for clinicians. COVID-19, caused by severe acute respiratory syndrome coronavirus 2 (SARS-CoV-2), is known to be associated with increased coagulopathy and thrombotic complications [165]. Current data are insufficient to make evidence-based recommendations related to the ITP management. Pavord et al. published a series of recommendations based on expert opinion on the management of ITP during the COVID-19 pandemic [166]. They drew attention to a possible further increased risk of thrombosis in patients with COVID-19 from ITP or its treatment (particularly with TPO-RA). Mahevas et al. reported in a case series that COVID-19-associated ITP can lead to profound thrombocytopenia and severe bleeding manifestations but has a favourable outcome in most cases [167]. More studies are needed to make evidence-based decisions on managing ITP during the pandemic.

Current guidelines state that patient preferences should be prioritized when choosing a treatment regimen. Important factors that determine patient preferences include treatment efficacy and the potential for complications. Efficacy and safety data from post-marketing studies of new treatments will be helpful in this regard. In addition, randomized controlled trials comparing existing treatments not only in terms of treatment response or safety but also in terms of their impact on the health-related quality of life of patients with ITP are needed.

## Figures and Tables

**Table 1:** Treatment options for ITP.

Agent	Application Route and Dosage	Advantages	Disadvantages and Complications
First-line Therapies			
Glucocorticoids			
Predniso(lo)ne	Oral 1 mg/kg of body weight for 2–3 weeks (maximum 80 mg/d), gradual tapering	Response within 1–2 weeks Early response rate 60–80%	Low durable response rate after discontinuation (30–50%) Complications: hypertension, hyperglycaemia, sleep and mood disturbances, gastric ulceration, myopathy, glaucoma and osteoporosis
Dexamethasone	Oral 40 mg for 4 days Maximum 3 cycles		
Immunoglobulin	Intravenous 0.4–1 gr/kg of body weight, total maximal dose of 2 gr/kg of body weight	Response within 1–4 days Early response rate 70–80%	Only a transient rise of platelet count Complications: headache, pyrexia, vomiting, acute kidney injury, aseptic meningitis and thrombotic events
Anti-Rhesus D Ig	Intravenous 50 to 75 µg/kg	Early response rate 65%	Only effective in Rh-positive patients Not approved for ITP in Europe Complications: headache, nausea, chills, fever and mild to moderate haemolysis Severe intravascular haemolysis and disseminated intravascular coagulation
Second-line Therapies			
Thrombopoietin-receptor Agonists			
Romiplostim	Subcutaneous 1–10 microg/kg/week	Response rate 70–80% Remission rate 10–30%	Cost Headache, arthralgia, myalgia, dizziness and insomnia Thromboembolism and bone marrow fibrosis
Eltrombobag	Oral 25–75 mg/day	Response rate 70–80% Remission rate 10–30%	Cost Dietary restrictions Gastrointestinal symptoms (nausea, vomiting, diarrhoea), mild transaminase elevations and headache Thromboembolism and bone marrow fibrosis
Avatrombopag	Oral 20–40 mg/day	Response rate 60% No dietary restrictions	Cost Headache, arthralgia, fatigue and diarrhoea
Immunomodulators			
Rituximab	Intravenous 375 mg/m^2^ per week for 4 weeks 100 mg/m^2^ per week for 4 weeks	Response rate 60% at 6 months No need for chronic treatment	High relapse rate Contraindicated by patients with evidence of an active or previous HBV infection Increased tendency to minor infections; progressive multifocal leukoencephalopathy
Fostamatinib	Oral 100–150 mg twice daily	Response rate 43% within 12 weeks after treatment	Diarrhoea, hypertension and nausea Monthly follow up for hypertension, hepatotoxicity and neutropenia.
Splenectomy	Open or laparoscopic surgery	Durable remission rate 60 to 70% No need for chronic treatment	Surgical complications, thromboembolic events, infection with encapsulated bacteria, Sepsis

## Data Availability

Not applicable.
